# Social inequalities in stage at diagnosis of rectal but not in colonic cancer: a nationwide study

**DOI:** 10.1038/sj.bjc.6604215

**Published:** 2008-01-29

**Authors:** B L Frederiksen, M Osler, H Harling, on behalf of Danish Colorectal Cancer Group, T Jørgensen

**Affiliations:** 1 Research Centre for Prevention and Health, Glostrup University Hospital, 2600 Glostrup, Denmark; 2 Department of Social Medicine, University of Copenhagen, 1014 Copenhagen K, Denmark; 3 Department of Surgery K, Bispebjerg University Hospital, 2400 Copenhagen NV, Denmark

**Keywords:** colorectal cancer, stage, socioeconomic status, inequality, nationwide

## Abstract

We investigated stage at diagnosis in relation to socioeconomic status (SES) among 15 274 patients with colorectal adenocarcinoma diagnosed in 1996–2004 nationwide in Denmark. The effect of SES on the risk of being diagnosed with distant metastasis was analysed using logistic regression models. A reduction in the risk of being diagnosed with distant metastasis was seen in elderly rectal cancer patients with high income, living in owner–occupied housing and living with a partner. Among younger rectal cancer patients, a reduced risk was seen in those having long education. No social gradient was found among colon cancer patients. The social gradient found in rectal cancer patients was significantly different from the lack of association found among colon cancer patients. There are socioeconomic inequalities in the risk of being diagnosed with distant metastasis of a rectal, but not a colonic, cancer. The different risk profile of these two cancers may reflect differences in symptomatology.

Differences in colorectal cancer mortality between socioeconomic groups are well documented (Auvinen, 1992; Kato *et al*, 1992; Monnet *et al*, 1993; Pollock and Vickers, 1997; Wrigley *et al*, 2003; Shaw *et al*, 2006). As stage is the major determinant of long-term mortality from colorectal cancer, its relation to socioeconomic status (SES) is of particular relevance. In a society like Denmark, with equal access to health-care facilities, SES might influence stage at diagnosis by psychosocial pathways. Thus, high-SES people may have more rational health behaviour, be more aware of their symptoms and communicate better with health staff than low-SES people. In consequence, the former may have a higher chance of early cancer detection. This, together with the different symptoms of cancer in the rectum and colon, led us to investigate the relation between SES and stage in these cancers separately. To our knowledge, only one previous study has done so but had some statistical limitations and used a rather crude measure of SES, namely county-level poverty (Wu *et al*, 2006). Two other studies of colon cancer alone found no statistically significant social gradient in the risk of late-stage disease (Auvinen, 1992; Vineis *et al*, 1993).

In this study, we investigated the influence of various SES indicators on stage at diagnosis of colon and rectum cancers in a nationwide Danish study.

## MATERIALS AND METHODS

The study population was derived from the Danish Rectal Cancer Database and the Danish Colorectal Cancer Database, which include about 93% of patients diagnosed in Denmark with a first-time adenocarcinoma of the rectum or rectum and colon, respectively (Harling *et al*, 2004; DCCG, 2006). A total of 4700 patients, diagnosed between 1 January 1995 and 31 August 1999, were from the Rectal Database, and 12 236 patients, diagnosed between 1 May 2001 and 31 December 2004, from the Colorectal Database. Throughout the year 2000, when the Rectal Database was transferred to the Colorectal Database, no patients were registered. From the latter, 39 patients were excluded – 16 due to missing information, one underage (10 years), one due to incorrect registration date and 21 were already in the Rectal Database. Among the remaining 16 897 patients, 1382 (8%) had not been Dukes classified and were excluded as were 216 (1%) due to missing socioeconomic variables leaving a total of 15 299 patients for analysis, of which 139 had a malignant polyp.

Patients classified with distant metastasis included those registered with distant metastasis in the Dukes classification or with spread to the peritoneum or metastasis to lungs or liver recorded in diagnostic or operative variables. The data were entered in the databases from questionnaires sent to the surgeons; it covered tumour stage, diagnostic procedures, localisation and mode of operation.

The socioeconomic data were derived by data linkage to the population-based integrated database for Labour Marked Research (IDA) administered by Statistics Denmark since 1980. Variables in the database are based on linkage between all inhabitants in Denmark (5.4 million per January 2004), all companies with more than one employee (around 230 000), the taxation authorities, the Registry Relating to Unemployment, the Integrated Student Registry and the Central Population Registry (Statistics Denmark, 1991).

Gross income, comprising all income subject to income taxation (wages and salaries, all types of benefits and pensions), was obtained for each patient and cohabiting partner. Income was corrected for inflation using Statistics Denmark's price index. Yearly variation was accounted for by calculating the average income in the 5 years before the diagnosis. For patients living with a partner, the average income of both was calculated. Income was grouped in quintiles for each strata of age.

Education was categorised into three groups: short education (corresponding obligatory education of 7 and 9 years for patients born before and after 1 January 1958, respectively), medium education (between 8/10 and 12 years – secondary school and vocational education) and higher education (above 12 years). Information on education was not available for persons born before 1920.

Employment was represented by the variable ‘SOCIO’, which is generated from several registers and specifies the character of the most important employment during the year before diagnosis. Because the most people aged above 65 years are retired, employment was considered relevant only under this age. The variable was categorised into three groups: ‘out of workforce’ (consisting of those unemployed, on invalidity pension, voluntarily early retired and five who were still receiving education), ‘wage earners – basic level’ (in jobs requiring basic skills and self-employed with no employee) and ‘wage earners – higher level’ (in jobs requiring higher skills, including managers, and self-employed with one or more employees).

Cohabitation status was categorised as ‘single’ and ‘living with partner’. Housing status was categorised as ‘owner–occupied’ and ‘rental’.

### Statistical methods

The main outcome was defined as the presence of distant metastases, and analyses were performed for colonic and rectal cancer separately. Logistic regression was used to examine the influence of each factor on the outcome using the procedure PROC LOGISTIC in SAS 9.1. A two-step model was used. In the baseline model, each socioeconomic variable was entered alone and adjusted for age and sex. The rectal cancer data were also adjusted for period (1995–1999 *vs* 2001–2004). County of residence and tumour localisation in the right or left side of the colon were removed from the model, as these factors had no or only marginal effect on the estimates. Analyses were stratified at 65 years of age (the typical age of retirement) when income decreases substantially and because the SES variable of employment was not relevant in the elderly. Furthermore, there was a statistically significant interaction of the effect of housing status and age on colonic cancer stage. No other interactions of age or sex and SES variables were seen. Two age groups, ‘younger’ and ‘elderly’, were considered. Test for trend was performed where relevant. Linearity of age was assured, but in elderly rectal cancer patients, the square of age showed the best fit. In the fully adjusted model, analyses were performed by entering all the socioeconomic variables. Because there were only minor differences in the estimates between the two models, only data of the fully adjusted model are shown in Tables 3 and 4. For evaluating a possible difference in effect of SES on stage in the two cancer sites, an interaction term (e.g., income × site) was included in analyses of the total population of elderly patients. A *P*-value <0.05 was used as level of significance.

## RESULTS

Of the 15 299 patients, 7725 had rectal and 7574 colonic cancer, with mean age of 68 and 71 years, respectively. Clinical and socioeconomic characteristics are shown in Tables 1 and 2. In the four strata, the frequencies of distant metastasis varied from 20 to 26%. Unadjusted analyses showed no major differences in the distribution of socioeconomic variables between rectal and colonic cancer, except a slightly higher frequency of elderly rectal cancer patients with low income, living with a partner and in owner–occupied housing as compared to corresponding colonic cancers. The mean and median income in the younger group was 273 507 and 249 912 Danish kroner (dkkr), respectively, and in the elderly group 162 197 and 129 248 dkkr, respectively.

The fully adjusted analysis showed that the risk of being diagnosed with distant metastases of rectal cancer decreased by age among the younger patients, but increased in the elderly patients until 75 years of age, after which it decreased (Table 3). In both age groups, an association was found between sex and stage (reduced risk in women) – although not significant. In the elderly patients, the risk of distant metastases was substantially and significantly reduced with increasing income, whereas in the younger patients there was no consistent association. Higher education significantly reduced the risk as compared to short education among the younger patients, while no influence of educational level was found among the elderly patients. Among the younger patients, a significant trend of employment was seen in the baseline model, but disappeared after adjustment for the other socioeconomic variables, the effect of employment being mediated by income (data not shown). In the elderly patients, a significant higher risk of distant metastases was seen for those living in rental housing as compared to owner–occupied housing and for those living alone *vs* living with a partner. No influence of cohabiting and housing status was seen in the younger patients.

In colonic cancer, women had a reduced risk of being diagnosed with distant metastases as compared to men (Table 4). The risk of distant metastases of a colonic cancer was not influenced by age, income, education, housing or cohabiting status, and also employment did not influence the risk among the younger patients. The impact of income, housing status and cohabiting on stage at diagnosis among the elderly patients with rectal cancer differed significantly from the lack of association found in the elderly patients with colonic cancer (interaction between cancer site and income, housing status and cohabiting status in the total elderly population: *P*=0.011, 0.001 and 0.031, respectively).

## DISCUSSION

This Danish study of nearly all patients diagnosed with colon and rectum cancer showed an association between SES and stage at diagnosis of rectal but not of colonic cancer. Rectal cancer showed a higher risk of distant metastases in the elderly patients with low income, rental housing and living single, while the younger patients with short education also had a higher risk of distant metastases. In colonic cancer, only sex had an influence on the risk of distant metastases.

The social inequality in rectal but not colonic cancer, to some extent, accords with a large American study, to the best of our knowledge, the only other study of SES and stage stratified by location (Wu *et al*, 2006). This study found a higher risk of late-stage disease in rectal and distal colonic cancer patients living in high-poverty counties as compared to low-poverty counties, and no social gradient in proximal colonic cancer patients. No statistical test, though, was applied to verify the significance of the difference between the SES groups. Two other studies restricted to colonic cancer found no significant socioeconomic inequalities in relation to stage (Auvinen, 1992; Vineis *et al*, 1993). Other studies have examined colonic and rectal cancers together, making comparison with the present study less relevant.

In our study, there was a general trend (although not significant in all subgroups) for male patients to show increased risk of distant metastases as compared to female patients, in both rectal and colonic cancers. This may reflect greater awareness of symptoms among women, especially from the lower abdomen, and the fact that women do visit doctors more regularly than men (The National Health Insurance Service Registry, 2007) .

The stronger influence of SES on stage in rectal than in colonic cancer may be due to differences in their symptoms. Clinical experience and studies indicate that the commonest initial symptoms of colonic cancer are vague and confusable with those of irritable colon, whereas initial symptom of rectal cancer is often rectal bleeding (Danish Centre for Health Technology Assessment (DACEHTA), 2001; Korsgaard *et al*, 2006b). Thus, it is more difficult for individuals, regardless of SES, to react early to the symptoms of a colonic than that of rectal cancer. Also relevant could be the positive association between diagnostic delay and late-stage disease for rectum, but not colon, cancer found in a study mainly consisting of patients in the Danish Colorectal Cancer Database (and thus overlapping with the present study) (Korsgaard *et al*, 2006a). If differing biology of cancer in different social groups was relevant, one would have expected to find social inequality in both rectal and colonic cancer patients.

We measured SES by several measures (income, education, cohabitation, housing conditions and employment), and could thereby encompass SES from slightly different angles. This is important, as these parameters are not always correlated and can have different implications on health (Geyer *et al*, 2006). Higher income may translate into increased access to health information and better resources to master stressful and demanding situations by seeking professional help. In Denmark, a direct economic hindrance in seeking medical health care cannot be relevant, because health-care facilities are tax-financed. It could, however, explain social inequalities found in stage of colorectal cancer in the United States (unstratified analyses of colorectal cancer) (Mandelblatt *et al*, 1996; Parikh-Patel *et al*, 2006). Education may influence awareness and ability to react practically, for instance how to manage symptoms of disease, and the ability to be insisting or demanding in the contact to the health-care system if necessary (Geyer *et al*, 2006). However, in the elderly patients, among whom other skills have developed over a lifetime, educational status may not be a good SES indicator (Galobardes *et al*, 2006), as implied in our data. Housing status reflects the cumulative influence of income throughout the life course (Avlund *et al*, 2003). Further, people who own their housing in old age are likely to be healthier and more active than people who have had to move to rental housing. Also, cohabiting status may influence health-related behaviour (Umberson, 1992; Johansen *et al*, 1996; Schone and Weinick, 1998), and be reflected in our data. Employment reflects an individual's place in society and how their education is used. This variable showed a protective trend against distant metastasis by increasing level of employment in the younger rectal cancer patients, in the first model, but in the mutually adjusted model, the effect ‘disappeared’, as it was mediated through income (data not shown).

The strength of this study is that it uses individual SES data from a nationwide study, and thus unselected population with colon or rectum cancer thereby avoiding misclassification, which is often seen when using area-based indicators of SES (McLoone and Ellaway, 1999). Further, all information on SES is collected prospectively and only for administrative purpose, thus eliminating recall bias. Another advantage is the separate analysis of colon and rectum cancers.

A limitation is that 8% of the primary population was excluded, because they had no Dukes’ classification, which may have induced differential bias, as univariate *χ*^2^ test revealed that they were significantly older and of lower SES as compared to classified patients. If these patients had been classified as diagnosed without distant metastases, it would have decreased the social gradient in relation to stage and vice versa.

For the purpose of this study, it should be remarked that screening programmes have not been offered to the Danish population in the study period. Potentially, screening could contribute to an accentuation of a social gradient in stage in both cancers, because primarily high-SES persons join screening programmes (McCaffery *et al*, 2002; Whynes *et al*, 2003; Singh *et al*, 2004).

In conclusion, this population-based study showed social inequality in stage at diagnosis in rectal cancer but not in colonic cancer. This may reflect the different symptomatology of cancers at the two sites. Only with definite symptoms, as is seen more frequently in rectal cancer, the patient has a chance to use skills, associated with SES, to diminish delay and thereby stage. Our findings indicate that information campaigns should be addressed especially towards low-SES people and focus on symptomatology.

## Figures and Tables

**Table 1:** Descriptive characteristics of 7725 rectal cancer patients diagnosed in Denmark, 1995–1999 and 2001–2004, according to age groups and cancer stage

	**Rectum**			
	**Age 18–64 years 36% (*N*=2762)**		**Age 65+ years 64% (*N*=4963)**	
	**Dukes ABC 80% (*N*=2206)**	**Distant metastases 20% (*N*=556)**	**Dukes ABC 78% (*N*=3859)**	**Distant metastases 22% (*N*=1104)**
**Characteristics**	**% (*N*)**	**% (*N*)**	**% (*N*)**	**% (*N*)**
*Age at diagnosis (years)*				
<34	1 (25)	2 (10)	—	—
35–44	5 (102)	7 (37)	—	—
45–54	26 (582)	28 (154)	—	—
55–64	68 (1497)	64 (355)	—	—
65–74	—	—	51 (1983)	48 (533)
75–84	—	—	41 (1574)	43 (476)
>85	—	—	8 (302)	9 (95)
				
*Sex*				
Female	41 (903)	38 (211)	42 (1637)	42 (459)
Male	59 (1303)	62 (345)	58 (2222)	58 (645)
				
*Year of diagnosis*				
1995–1999	53 (1169)	53 (292)	53 (2059)	52 (569)
2001–2004	47 (1037)	47 (264)	47 (1800)	48 (535)
				
*Dukes’ classification*				
A (incl. malignant polyp)	17 (463)	—	17 (827)	—
B	30 (827)	—	32 (1588)	—
C	33 (916)	—	29 (1444)	—
Distant metastases	—	20 (556)	—	22 (1104)
				
*Gross income*				
First quintile – lowest	21 (467)	18 (101)	22 (845)	26 (292)
Second quintile	20 (436)	24 (136)	19 (718)	20 (226)
Third quintile	20 (434)	21 (115)	19 (743)	19 (211)
Fourth quintile	20 (443)	19 (107)	20 (761)	19 (204)
Fifth quintile – highest	19 (426)	18 (97)	20 (792)	16 (171)
				
*Highest education*				
Short	27 (589)	29 (163)	34 (1307)	34 (378)
Medium	51 (1124)	52 (289)	27 (1054)	26 (284)
Higher	20 (438)	16 (86)	9 (344)	8 (88)
Unknown	2 (55)	3 (18)	30 (1154)	32 (354)
				
*Employment*				
Out of workforce	36 (794)	38 (211)		
Wage earners – basic level	40 (878)	40 (222)		
Wage earners – higher level	24 (534)	22 (123)		
				
*Cohabiting*				
Single	23 (511)	23 (128)	40 (1565)	46 (509)
Living with partner	77 (1695)	77 (428)	60 (2294)	54 (595)
				
*Housing status*				
Owner–occupied	71 (1574)	69 (383)	57 (2202)	50 (551)
Rental	29 (632)	31 (173)	43 (1657)	50 (553)

**Table 2:** Descriptive characteristics of 7574 colonic cancer patients diagnosed in Denmark, 2001–2004, according to age groups and cancer stage

	**Colon**			
	**Age 18–64 years 27% (*N*=2044)**		**Age 65+ years 73% (*N*=5530)**	
	**Dukes ABC 74% (*N*=1501)**	**Distant metastases 26% (*N*=543)**	**Dukes ABC 77% (*N*=4269)**	**Distant metastases 23% (*N*=1261)**
**Characteristics**	**% (*N*)**	**% (*N*)**	**% (*N*)**	**% (*N*)**
*Age at diagnosis (years)*				
<34	1 (20)	2 (11)	—	—
35–44	6 (83)	7 (36)	—	—
45–54	25 (372)	23 (125)	—	—
55–64	68 (1026)	68 (371)	—	—
65–74	—	—	42 (1785)	42 (535)
75–84	—	—	45 (1926)	46 (583)
>85	—	—	13 (558)	11 (143)
				
*Sex*				
Female	49 (737)	44 (238)	54 (2290)	49 (613)
Male	51 (764)	56 (305)	46 (1979)	51 (648)
				
*Year of diagnosis*				
1995–1999	—	—	—	—
2001–2004	100 (1501)	100 (543)	100 (4269)	100 (1261)
				
*Dukes’ classification*				
A (incl. malignant polyp)	9 (179)	—	8 (457)	—
B	34 (689)	—	42 (2300)	—
C	31 (633)	—	27 (1512)	—
Distant metastases	—	26 (543)	—	23 (1261)
				
*Gross income*				
First quintile – lowest	19 (288)	22 (117)	17 (746)	17 (212)
Second quintile	20 (293)	16 (88)	21 (905)	22 (274)
Third quintile	21 (309)	20 (108)	21 (877)	21 (263)
Fourth quintile	19 (292)	20 (108)	21 (879)	20 (251)
Fifth quintile – highest	21 (319)	22 (122)	20 (862)	20 (261)
				
*Highest education*				
Short	25 (372)	22 (119)	36 (1524)	38 (485)
Medium	52 (784)	55 (297)	30 (1269)	30 (373)
Higher	22 (326)	21 (114)	10 (416)	9 (117)
Unknown	1 (19)	2 (13)	25 (1060)	23 (286)
				
*Employment*				
Out of workforce	35 (528)	35 (189)		
Wage earners – basic level	41 (608)	42 (231)		
Wage earners – higher level	24 (365)	23 (123)		
				
*Cohabiting*				
Single	23 (352)	26 (143)	47 (2025)	48 (600)
Living with partner	77 (1149)	74 (400)	53 (2244)	52 (661)
				
*Housing status*				
Owner–occupied	73 (1103)	69 (373)	52 (2200)	52 (661)
Rental	27 (398)	31 (170)	48 (2069)	48 (600)

**Table 3:** Mutually adjusted odds ratios (including 95% CI) of distant metastases in 7725 rectal cancer patients diagnosed in Denmark, 1995–1999 and 2001–2004, according to age groups

	**Rectum**	
	**Age 18–64 years**	**Age 65+ years**
*N* (distant metastasis/Dukes ABC)	556/2206	1104/3859
	Fully adjusted	Fully adjusted
*Age*		
Age (per year)	0.98 (0.96–0.99)	—a
*P*-value	0.0016	0.049
		
*Sex*		
Male	1	1
Female	0.84 (0.69–1.02)	0.88 (0.76–1.01)
*P*-value	0.0837	0.0867
		
*Gross income: quintiles*		
First quintile – lowest	0.72 (0.48–1.06)	1.61 (1.27–2.05)
Second quintile	1.14 (0.81–1.60)	1.38 (1.08–1.76)
Third quintile	1.03 (0.75–1.43)	1.29 (1.01–1.60)
Fourth quintile	1.00 (0.73–1.37)	1.27 (1.01–1.60)
Fifth quintile – highest	1	1
*P*-value	0.0624	0.0038
*P*-value – trend		0.0002
		
*Highest education*		
Short	1	1
Medium	0.87 (0.70–1.09)	1.03 (0.86–1.24)
Higher	0.66 (0.47–0.93)	1.14 (0.86–1.52)
Unknown/born before 1920	1.17 (0.66–2.05)	1.09 (0.86–1.39)
*P*-value	0.0779	0.7651
*P*-value – trend	0.0190^b^	
		
*Employment*		
Out of workforce	1.31 (1.02–1.70)	
Wage earners – basic level	1	
Wage earners – higher level	1.07 (0.81–1.43)	
*P*-value	0.1166	
		
*Housing status*		
Owner–occupied	1	1
Rental	1.15 (0.91–1.44)	1.21 (1.05–1.40)
*P*-value	0.2348	0.0097
		
*Cohabiting*		
Living with partner	1	1
Single	0.95 (0.75–1.20)	1.28 (1.09–1.50)
*P*-value	0.6551	0.0021

CI, confidence interval.

*P*-values calculated as Wald *χ*^2^.

a Age was modulated as age square in this stratum. Degrees of freedom=2.

b Test for trend, when excluding 73 patients with unknown education.

**Table 4:** Mutually adjusted odds ratios (including 95% CI) of distant metastases in 7574 colonic cancer patients diagnosed in Denmark, 2001–2004, according to age groups

	**Colon**	
	**Age 18–64 years**	**Age 65+ years**
*N* (distant metastasis/Dukes ABC)	543/1501	1261/4269
	Fully adjusted	Fully adjusted
		
*Age*		
Age (per year)	1.00 (0.98–1.01)	1.00 (0.98–1.01)
*P*-value	0.5185	0.4628
		
*Sex*		
Male	1	1
Female	0.82 (0.67–1.00)	0.78 (0.68–0.89)
*P*-value	0.0481	0.0003
		
*Gross income: quintiles*		
First quintile – lowest	0.94 (0.64–1.40)	0.92 (0.73–1.17)
Second quintile	0.73 (0.51–1.05)	1.00 (0.81–1.24)
Third quintile	0.85 (0.61–1.19)	0.96 (0.78–1.18)
Fourth quintile	0.91 (0.66–1.25)	0.93 (0.76–1.13)
Fifth quintile – highest	1	1
*P*-value	0.4307	0.9661
		
*Highest education*		
Short	1	1
Medium	1.17 (0.90–1.52)	0.89 (0.76–1.05)
Higher	1.13 (0.79–1.60)	0.82 (0.64–1.06)
Unknown/born before 1920	2.09 (1.00–4.37)	0.87 (0.70–1.09)
*P*-value	0.2225	0.3028
		
*Employment*		
Out of workforce	0.95 (0.73–1.25)	
Wage earners – basic level	1	
Wage earners – higher level	0.83 (0.62–1.13)	
*P*-value	0.5051	
		
*Housing status*		
Owner–occupied	1	1
Rental	1.24 (0.97–1.58)	0.98 (0.85–1.12)
*P*-value	0.0896	0.7306
		
*Cohabiting*		
Living with partner	1	1
Single	1.09 (0.85–1.38)	1.11 (0.96–1.30)
*P*-value	0.5084	0.1716

CI, confidence interval.

*P*-values calculated as Wald *χ*^2^.
